# Communicating prognostic uncertainty in potential end-of-life contexts: experiences of family members

**DOI:** 10.1186/s12904-016-0133-4

**Published:** 2016-07-12

**Authors:** Marian Krawczyk, Romayne Gallagher

**Affiliations:** Centre for Health Evaluation and Outcome Sciences, St. Paul’s Hospital, 588 – 1081 Burrard Street, Vancouver, BC V6Z 1Y6 Canada; Division of Palliative Care, UBC, Department of Family and Community Medicine, Providence Health Care, 1081 Burrard St., Vancouver, BC V6Z 1Y6 Canada

**Keywords:** Prognostic uncertainty, End of life, Family members, Communication

## Abstract

**Background:**

This article reports on the concept of “communicating prognostic uncertainty” which emerged from a mixed methods survey asking family members to rank their satisfaction in seven domains of hospital end-of-life care.

**Methods:**

Open-ended questions were embedded within a previously validated survey asking family members about satisfaction with end-of-life care. The purpose was to understand, in the participants’ own words, the connection between their numerical rankings of satisfaction and the experience of care.

**Results:**

Our study found that nearly half of all family members wanted more information about possible outcomes of care, including knowledge that the patient was “sick enough to die”. Prognostic uncertainty was often poorly communicated, if at all. Inappropriate techniques included information being cloaked in confusing euphemisms, providing unwanted false hope, and incongruence between message and the aggressive level of care being provided. In extreme cases, these techniques left a legacy of uncertainty and suspicion. Family members expressed an awareness of both the challenges and benefits of communicating prognostic uncertainty. Most importantly, respondents who acknowledged that they would have resisted (or did) knowing that the patient was sick enough to die also expressed a retrospective understanding that they would have liked, and benefitted, from more prognostic information that death was a possible or probable outcome of the patient’s admission. Family members who reported discussion of prognostic uncertainty also reported high levels of effective communication and satisfaction with care. They also reported long-term benefits of knowing the patient was sick enough to die.

**Conclusion:**

While a patient who is sick enough to die may survive to discharge, foretelling with family members in potential end of life contexts facilitates the development of a shared and desired prognostic awareness that the patient is nearing end of life.

## Background

National and international initiatives for improving end-of-life care highlight the need to meaningfully engage family members, including their concerns regarding satisfaction with care. Specialist palliative care has traditionally been championed as the best way to ensure family member satisfaction with end-of-life care, yet research evidences that such a model alone cannot result in significant population or system-level improvements [1]. This is in part due to increasing prognostic uncertainties caused by multi-morbidity and/or new treatment possibilities for advancing chronic life-limiting illness. Negotiating prognostic uncertainty with this growing population is a particular challenge for hospital-based clinicians as these patients are also often frail, near the end stage of their illness(es), and are admitted due to an acute event and/or poorly controlled exacerbation. These patients also have a high incidence of rapid deterioration and the care outcome during any single hospital admission is often death. However, with medicine’s increasing ability to “rescue” this patient population from acute episodes even as they approach death, prognostication remains challenging. While acute interventions can trouble clear clinical indictors of end of life with these patients, they can be understood as “sick enough to die” [2]. There is no mutually agreed upon term for patients whose death is not clinically unexpected at any time in the relatively near future–during admission, over the ensuing months, or occasionally, years. Other descriptors include “people who will die in the (relatively) near future” [2], patients at “high risk for mortality” [3], “ambiguous dying” [4], “persons who will die as a result of serious and complex illness” [5], or patients “who reasonability might die” [6]. We believe, in keeping with plain language, the term “sick enough to die” may have the most meaning for the average individual.

The lack of predictive certainty as to when death will occur is cited as a key reason in hospital-based clinicians’ (HBC) high rates of self-reported unwillingness to introduce or discuss the possibility of dying [7–10]. Other research highlights that while HBC may experience initial prognostic uncertainty about these patients upon admission, they also reported knowledge of impending death several days before it occurred [11, 12]. Consequently, prognostic uncertainty–even as it may resolve over the course of admission–offers a powerful justification for not communicating with family members that a patient is sick enough to die, until the patient is actively dying. Little remains known about family members’ perspectives about communicating prognostic uncertainty in potential end of life contexts [13]. In this paper we evidence how communicating prognostic uncertainty impacts family members, and explore why prognostic forecasts should focus on raising awareness that a patient is sick enough to die. We do so through describing family members’ self-reported experiences of HBCs’ communication regarding prognostic uncertainty in acute care settings where the patient was sick enough to–and did–die.

## Methods

As part of a strategy to improve access and build capacity for palliative care across Providence Health Care (Vancouver, British Columbia), we undertook a post-mortem telephone audit with 90 relatives of patients four to six months after the patient’s death, and focusing on the last 48 h of care, including hospital, hospice, and residential care settings. Next of kin records were drawn from all patient deaths over a period of seven months in 2009 and 2010. Patients who were under the age of 18, died from trauma, or who died before being admitted into the hospital were excluded. From the 553 potential participants, 225 entries were invalidated due to: 1) in care less than 48 h; 2) next of kin and emergency contact outside of province, or 3) no contact or incomplete information. An invitational letter was mailed to the remaining 332 potential participants. If they were thought to speak either a Chinese language or Punjabi, they were sent an accompanying letter in that language stating the interview could be conducted through a professional interpreter. Ten business days later, those who did not call the refusal phone number provided were contacted by the interviewer for a total of three attempts over a one-month period. The interviewer [MK] assessed inclusion and exclusion criteria and arranged to conduct the interview. Participants had to speak English, Cantonese, Mandarin or Punjabi and attended to the patient during the last weeks of the patient’s life as well as had experience with the patient’s health care providers in the last forty-eight hours of life. Bereaved relatives who did not meet the above criteria, were experiencing acute emotional distress, or were suspected of having cognitive issues such as dementia were excluded. Employees of Providence Health were also excluded. Ten potential participants were excluded by these criteria. Of the 292 remaining potential participants, 146 did not respond to phone messages, 30 could not be contacted from the information provided or had moved with no forwarding information, 35 declined to participate once contacted, 6 felt they were not well enough to participate, 4 stated the patient received good or excellent care but felt that discussing it further would be too painful, and 3 sent back consents but the interviewer was unable to contact them. A total of 90 interviews were conducted. Interviews lasted approximately 30 min (range 17–75 min). Informed consent was obtained for each participant.

Here we report only on findings specific to the hospital setting (*n* = 67) in order to further contextualize our overall findings that communication emerged as one of the two lowest rankings regarding satisfaction with care at the end of life. The After-Death Bereaved Family Member Interview is a validated multi-domain survey tool that examines seven domains of end-of-life care, including:

- Physical and emotional support
- Inform and promote shared decision-making
- Encourage advance care planning
- Focus on individual
- Attend to the emotional and spiritual needs of the family
- Provide coordination of care
- Support for the self-efficacy of the family

One overall domain ranking question, and two sub-measure questions that were part of the original quantitative survey addressed aspects of prognostic communication (see Table 1). The quantitative results of our study have been published elsewhere, including detailed patient demographics [14]. Our study added a question to each survey section that numerically assessed (on an 11-point Likert scale) family members’ overall satisfaction of care within an individual domain. The open-ended question asked participants to elucidate the reasons for their overall score in each domain, and was worded “Can you please tell me a bit more about why you gave this number?” The purpose was to understand, in the participants’ own words, the connection between the numerical ranking and the experience of care. The general anticipatory goal of including the open-ended question was to ensure experiential context for strengthening relevance of the quantitative findings.

**Table 1:** Survey responses for communication relevant to prognosis

Last place of care	Palliative Care Unit	Intensive Care Unit	General Acute	Total
Number of respondents	24 (36 %)	9 (13 %)	34 (51 %)	67 (100 %)
*In the last two days of care, how well did the doctors, nurses, and other professional staff communicate about with patient and family about the illness and likely outcomes of care?*				
Not satisfied	2 (8 %)	2 (22 %)	10 (29 %)	14 (20 %)
Satisfied/somewhat satisfied	6 (25 %)	0	5 (15 %)	11 (16 %)
Very satisfied	16 (67 %)	7 (78 %)	19 (56 %)	36 (54 %)
*In (patient’s) last two days of care, how often were you or other family members kept informed about (patient’s condition) – always, usually, sometimes, never?*				
Always	9 (37 %)	7 (78 %)	13 (38 %)	29 (43 %)
Usually, sometimes, or never	15 (63 %)	2 (22 %)	21 (62 %)	38 (57 %)
*Would you have wanted (some/more) information about what to expect while (he/she) was dying?*				
No	15 (62 %)	6 (66 %)	16 (47 %)	37 (55 %)
Yes	9 (38 %)	3 (33 %)	18 (53 %)	30 (45 %)

The interviewer took short-hand notes of the narrative responses, and transcribed them as closely to verbatim as possible immediately after each survey. What the authors had not anticipated (perhaps naïvely) was that family members predominantly wished to *talk* about their experiences of care, rather than merely assign numerical value to those experiences. Although we initially had no plans to engage in qualitative analysis, given the importance participants placed on being able to talk about their experiences, and the amount of narrative data generated, we felt further analysis could provide additional insights regarding bereaved family members’ experiences of end-of-life care. Grounded theory methods guided analysis of the narrative data [15]. Both authors independently read through the notes multiple times to inductively identify provisional codes. We then met, and using constant comparison method, compared and refined the codes to identify recurrent themes. We then refined these themes, grouping similar themes into clusters and thereby generated provisional conceptual categories. In this paper we focus on the category “communicating prognostic uncertainty” to describe one integrated, relational group of themes that emerged from the coding process. The use of an inductive approach to analysis allowed us to create an analytical framework based on participants’ self-reports, highlighting the importance and relevance of effective communication regarding prognostic uncertainty. The study was approved by Providence Health Care’s Research Ethics Board.

## Results

In keeping with the current trend in health care, the majority of hospitalized patients in our study were older (average age 76 years) and had multiple comorbidities, indicating that death was a possible outcome of hospital admission (average length of stay 22 days). Overall, most family member respondents were a child or spouse of the patient (85 %), and two-thirds were female, with an average age of 60 years. Nearly three-quarters (73 %) reported their ethno-cultural heritage as Canadian or European, more than half (60 %) reported a college or university degree, and 20 % stated their own health status as fair or poor.

From the quantitative results of our study, slightly more than 50 % of family members felt very satisfied with the quality of communication about the patient’s illness and likely outcomes of care. Less than half (43 %) reported that they were always kept informed about the patient’s condition, and close to half (45 %) reported wanting more information about what to expect about the patient’s dying process. All these percentages increased in units that did not have embedded specialist expertise in end-of-life care.

Our qualitative analysis identified ten themes in three clusters specific to family members’ reports of communication regarding uncertain prognostic outcomes of care. The first theme stands alone, and is based on reports of existing self-knowledge of the patient’s prognosis. The following five themes focus on the challenges family members identified as emerging from a lack of communication that the patient was sick enough to die until prognostic uncertainty resolved into active dying. The last four themes address family members’ self-reflexive understandings about the challenges and benefits of communicating and knowing the patient was sick enough to die, including the long-term benefits of knowing that the patient’s prognosis was uncertain.

### Theme one: Lack of awareness that patient was sick enough to die

Family members reported a lack of understanding that death was a possible outcome of the patient’s hospital admission, even as they expressed awareness that the patient was acutely ill. This was particularly true during the initial phase of admission.

*“Looking back on the situation I can see that he [patient] was going, but nobody told me so I wasn’t prepared”.*

*“It was hard because we were still being proactive in treating him and making plans for his future - he had recovered before.”*

### Theme two: Lack of communication about possible prognosis

Inextricably intertwined with the above theme was the concern that HBC did not discuss that the patient was potentially sick enough to die until the patient was actively dying.

*“The doctor knew it wasn't going to be positive. Instead of being straight up he wasn't; I never thought she was dying. He was very good on the last day, calling and saying we should come in.”*

*“They came in and looked after him but we were never really approached by anyone until the end - I would've liked to know how serious it was beforehand”.*

For some family members, this lack of communication about possible prognostic trajectory resulted in the belief that health care providers actively withheld information or knew something they did not.

*“I feel like the staff were trying to protect me. I wanted to know though so I could prepare… In some ways it was good because there was a family and team meeting, but they were never completely truthful and then there were clues that they didn’t explain to me. I thought he was rallying.”*

### Theme three: Dissonance between probable outcome of care and ongoing treatments

Family members also reported confusion about the acute care the patient continued to receive up to the point of death.

*“They were still giving him antibiotics, blood, plasma - he had a PICC line. It wasn't until the last night a doctor took me aside…He took me into the hall; it was the first time I'd heard that he wouldn't make the night. He was still getting all the treatment.”*

### Theme four: Inappropriate use of euphemisms

Family members also reported that health care providers who explained the patient’s medical situation through euphemisms caused distress, confusion, and uncertainty as to what to expect.

*“I didn't realize he was dying. He had a couple of scares and they called me and said that he wasn't doing too well and I said "what does that mean?" They just said "not well".*

*“He wasn't in the same state as they told me on the phone - they said he was 'comfortable' but he didn't seem to be. Resting comfortably really meant being comatose”.*

### Theme five: Provision of false hope

Family members reported that conversations about possible outcomes of admission that did not include discussion that the patient was sick enough to die generated an inappropriate “false hope”.

*“My brother asked about palliative care and the doctor said "oh no, we haven't given up on your mother", so I asked if my other brother [who lives away] should come. My instinct was yes, but on the phone with the doctor there was complete silence. Finally he said "if it were me, yes then I would tell him to come". I knew that the doctor was trying to build hope, but…I just needed the doctor to say…to tell me”.*

### Theme six: Suspicion of malfeasance

In not understanding that the patient was sick enough to die, several family members continued to question if something had been done incorrectly to cause death, leaving a legacy of uncertainty and suspicion.

*“I didn't know why he was breathing so heavily [a symptom common close to death] because nobody talked to me about it. It was like they thought I knew he was dying, but I didn't. Was his death natural? It wasn't a heart attack, and that's what they had prepared me for. Yes, he was very sick and it was a complicated case, but still.”*

Our analysis also identified four themes relating to self-reflexivity regarding the challenges and benefits of communicating uncertain prognosis. The first theme highlights an awareness that family members may resist “knowing” that a patient is sick enough to die.

*“We didn't want to hear that he was going to die, so the staff had to dance around that information while providing care…I didn't want to know. They were wary of saying anything because we didn't want to hear it.”*

The second theme addresses our most unexpected finding. Respondents who acknowledged that they would have expressed resistance (or did) to the knowledge that the patient might die, also expressed a retrospective understanding that they would have benefitted (or did) from knowing that death was a possible or probable outcome of the patient’s admission.

*“One nurse said "you know he is going to die", which completely snapped me out of my oblivion. I didn't appreciate it at the time but in retrospect I really did - it allowed me to prepare for the eventualities. I talked with the doctors and they thought he'd be fine just a few days before that”.*

The third theme centers on the impact of HBCs’ communication of prognostic uncertainty. Participants who reported that HBCs included prognostic uncertainty at some point in their communication also reported high levels of effective communication, adequate information sharing, and satisfaction with care.

*“The doctor was brilliant; fabulous. She spoke to all the family members, including my mother giving us all the information… Originally we were gearing towards a possible recovery so there was active treatment… [but] the doctor was clear near the end.”*

The final theme address reports that clear communication about uncertain care outcomes, ongoing communication as uncertainty resolved into active dying, and the ways this information was communicated, were powerful drivers as to how family members were able to spend the last days with the patient. This was conjoined with positive self-reports regarding the capacity to sit with the patient’s death, four to six months after its occurrence. This held true even if the death was perceived “a shock” and/or “unexpected” by family members.

*“If you're doing a study about this, tell them that family members need to know about the medicine and about death. Nobody wants to talk about it, but it's there…If that second doctor hadn't said to stay I would've gone [on vacation] and I would've deeply regretted it. I would have gone because of wrong information, so BE CLEAR.”*

## Discussion

Our study found that nearly half of all family members wanted more information about possible outcomes of care, including knowledge that the patient was “sick enough to die”. Many of these participants did not understand death was a possible outcome of the patient’s admission, even if they expressed an implicit, and at times explicit, awareness that the patient had been seriously ill for months, and in some cases, years. This seeming bifurcation of awareness is a common response to the uncertain prognostic trajectory for those living with advancing chronic life-limiting illnesses [16, 17]. Most Canadians currently die in a hospital setting from progression of their multi-morbidity. Consequently, hospital-based physicians become the primary source through which patients with advancing life-limiting illness(es) and their family members are informed of an anticipated, if temporally uncertain, death. Yet our study found that awareness of this prognostic uncertainty was often poorly communicated, if at all. Inappropriate techniques included information being cloaked in confusing euphemisms, providing unwanted false hope, and incongruence between message and the aggressive level of care being provided. In extreme cases, these techniques left a legacy of uncertainty and suspicion. We found that family members were cognisant of both the challenges and benefits of communicating prognostic uncertainty. Our most unexpected finding was respondents who acknowledged that they would have resisted (or did) knowing that the patient was sick enough to die also expressed a retrospective understanding that they would have liked, and benefitted, from more prognostic information that death was a possible or probable outcome of the patient’s admission. Family members who reported discussion of prognostic uncertainty also reported high levels of effective communication and satisfaction with care. They also reported long-term benefits of knowing the patient was sick enough to die, including a sense of peace and lack of regret in being able to spend time with patient before they were actively dying.

Uncertainty is a daily reality in medicine; yet we work diligently to eliminate it. Attempts to control prognostic uncertainty are done for patient benefit, as well as to demonstrate competence and professional behavior [18]. While a significant component of earning patient and family member trust is the open acknowledgement of prognostic uncertainty [13], acknowledgement can also be a source of mistrust and potential conflict [19]. Patients, family members and health care providers all report that communicating prognostic uncertainty is a desirable trait of person-centered care [8, 9, 20]. They also acknowledge that communication about prognosis requires discussion earlier in the disease trajectory, and sensitivity and awareness that it is a complex and individual process involving multiple discussions [21]. In relation to family members, our research echoes the few existing studies that find the majority of family members want clinicians to communicate that a patient is sick enough to die, understand that uncertainty is an unavoidable, that discussing uncertainty leaves room for hope, increases trust in physicians, and allows for self-preparation that the patient might not survive [13, 19, 20].

Prognosis is the skill of knowing and predicting the natural history of a disease based on the individual patient characteristics and possible complications. There are two parts to the process of prognostication: foreseeing and foretelling [22]. Foreseeing is the inward cognitive estimate of the future course of the patient’s illness. With experience and knowledge physicians will develop this skill (to a greater or lesser degree). Foretelling is the communication of this estimate to the patient and family. Similar to foreseeing, foretelling also requires time for skills to develop. Unlike foreseeing however, foretelling “bad news” can trigger substantial stress response and negative emotions for clinicians, including uncertainty, helplessness, and fears of death, iatrogensis, damage to reputation, and failure [23–25].

As hospital-based clinicians gain more experience, they also experience more uncertainty; a hallmark of an experienced clinician is their ability to tolerate uncertainty [26]. As new therapies increasingly reconfigure the course of chronic life-limiting diseases, however, clinical focus has prioritized diagnosis over prognosis [27, 28], and even experienced clinicians report significant challenges to their prognostic capacities [29]. Consequently, clinicians now report considerable stress related to their inability to tolerate high and protracted levels of ambiguity and/or uncertainty [25]. Clinicians working with patients who are near, or at end of, advancing chronic life-limiting illnesses may then avoid the emotional challenges that accompany the uncertainties in foretelling with this patient population by attributing their reticence to prognostic uncertainties that render them unable to adequately foresee. In turn, this discomfort, under the guise of the limits of medicine, often translates into the continued offering of aggressive therapies until the patient is actively dying. At the same time, however, the majority of patients “sick enough to die” report the desire for a treatment plan that focuses more on providing comfort than a technologically oriented, institutionalized death requiring repeated acute care admissions [30].

Practical certainty is being as certain as it is reasonable to be in the circumstances. This requires prognostication based on objective evidence of likely outcome, preferably involving the collective wisdom of a number of clinicians, and supported by the response of the patient to treatment over time [31]. When a patient near or at the end stage of one or more advancing chronic life-limiting illnesses is admitted to an acute care setting, physicians can be practically certain in foreseeing that these patients are “sick enough to die”. Yet they often do not translate this foreseeing into foretelling, mistakenly assuming that this latter skill is only required when they are certain death is immanent (often just hours before death).

Our research found that family members wanted hospital clinicians to translate practical certainty in foreseeing to foretell that the patient was “sick enough to die”, even though these conversations would be (or were) difficult. Participants who reported that health care providers included sensitive communication regarding prognostic uncertainty also reported high levels of satisfaction with care, even if the patient’s death was perceived as “a shock” and/or “unexpected” by family members. Our research with family members’ of patients “sick enough to die” supports existing research on prognostic communication in hospitals as an iterative process that needs to begin with a preliminary mention of the possibility of death early with this patient population, and becoming more detailed as the clinical situation develops.

### Limitations

The original purpose of attaching identical open-ended questions at the end of each overall ranking question was primarily to contextualize quantitative findings for ensuring relevant quality improvement initiatives internal to Providence Health. We therefore did not anticipate the need to audio-record family members’ narratives. Consequently, while the interviewer has extensive experience in qualitative interviewing, writing detailed ethnographic field notes, is trained in short-hand, and types 90 words a minute, we do not assume her notes represent complete fidelity. Both authors believe, however, that verbatim note-taking is a valid form of recording through which *reasonable* fidelity can be achieved, particularly in the generation of themes [32, 33]. Further, lack of formal audio-recording may have helped participants feel less constrained in their discussion of difficult or sensitive experiences [34].

The qualitative results of this study are based on responses to questions that did not directly ask participants to detail any specific experience of communication about prognostic uncertainty. Given that responses which included discussions of prognostic uncertainty were unstructured, we were not able to engage in any relevant statistical description, gender, or ethno-cultural analysis of those who specifically spoke about this issue. This limits applicability of the findings, in particular in relation to cultural groups that may have different cultural norms regarding the role of physicians and/or the communication of “bad news”.

## Conclusion

Patients with advancing chronic life-limiting illnesses are often hospitalized, and these hospitalizations increase as they near end of life. While a patient who is sick enough to die may survive to discharge, our research evidences family members’ desires for clinical foretelling in potential end of life contexts to facilitate the development, over time, of a shared and desired prognostic awareness that the patient is nearing end of life. To continue to treat an illness without willingness to foresee the future is to ignore a key competency of becoming a clinician. To continue to treat an illness without foretelling the future to the patient/family is not informed consent and robs people of their right to understand what may reasonably happen and thereby prepare. Foreseeing and foretelling are skills we need to master more than ever.

## Abbreviations

HBC, Hospital-based clinicians
